# Supplementary material for: Causal Relationship between Aging and Anorexia Nervosa: A White-Matter-Microstructure-Mediated Mendelian Randomization Analysis
Source: Biomedicines. 2024 Aug 16;12(8):1874. doi: 10.3390/biomedicines12081874 (PMC11351342; doi:10.3390/biomedicines12081874)
Supplement: Supplementary file 1 [file biomedicines-12-01874-s001.zip › Supplementary Figure/Figure S4/Singe SNP plot of MR analysis for TL to ubm-b-1629.pdf]

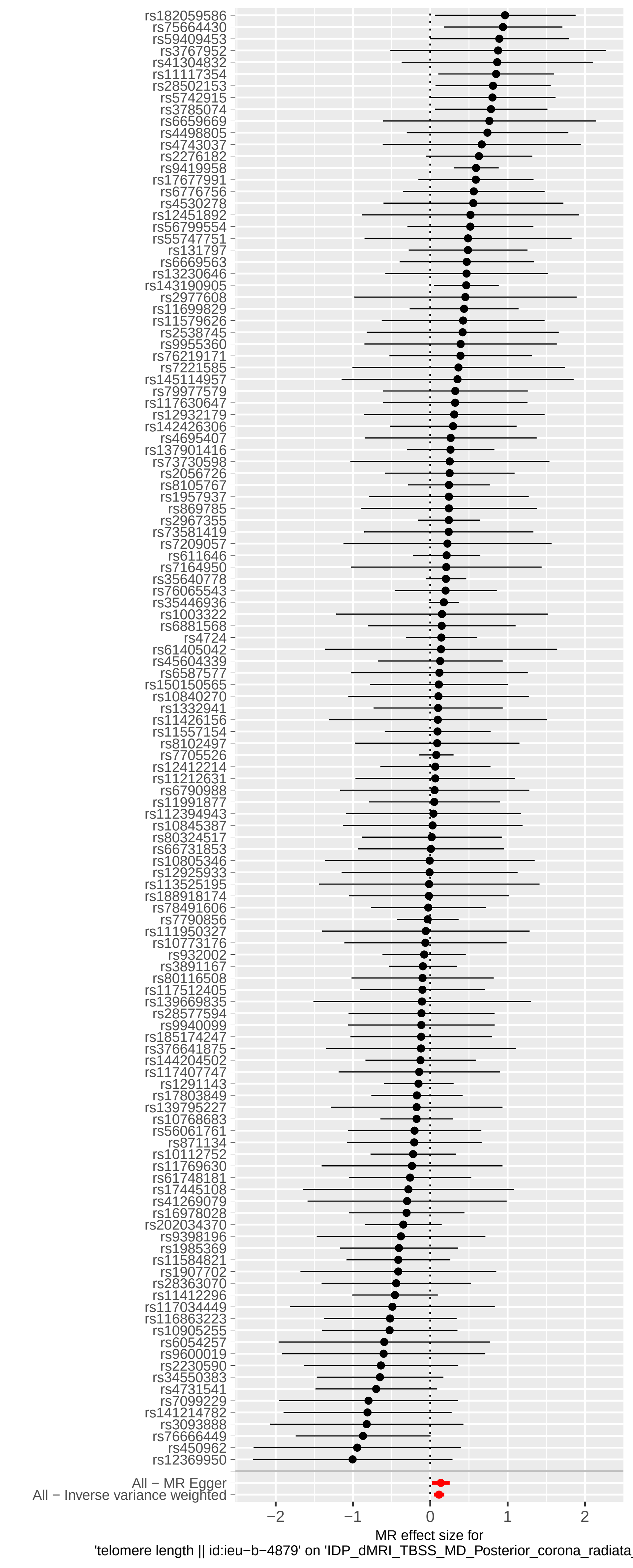
rs182059586
rs75664430
rs59409453
rs3767952
rs41304832
rs11117354
rs28502153
rs5742915
rs3785074
rs6659669
rs4498805
rs4743037
rs2276182
rs9419958
rs17677991
rs6776756
rs4530278
rs12451892
rs56799554
rs55747751
rs131797
rs6669563
rs13230646
rs143190905
rs2977608
rs11699829
rs11579626
rs2538745
rs9955360
rs76219171
rs7221585
rs145114957
rs79977579
rs117630647
rs12932179
rs142426306
rs4695407
rs137901416
rs73730598
rs2056726
rs8105767
rs1957937
rs869785
rs2967355
rs73581419
rs7209057
rs611646
rs7164950
rs35640778
rs76065543
rs35446936
rs1003322
rs6881568
rs4724
rs61405042
rs45604339
rs6587577
rs150150565
rs10840270
rs1332941
rs11426156
rs11557154
rs8102497
rs7705526
rs12412214
rs11212631
rs6790988
rs11991877
rs112394943
rs10845387
rs80324517
rs66731853
rs10805346
rs12925933
rs113525195
rs188918174
rs78491606
rs7790856
rs111950327
rs10773176
rs932002
rs3891167
rs80116508
rs117512405
rs139669835
rs28577594
rs9940099
rs185174247
rs376641875
rs144204502
rs117407747
rs1291143
rs17803849
rs139795227
rs10768683
rs56061761
rs871134
rs10112752
rs11769630
rs61748181
rs17445108
rs41269079
rs16978028
rs202034370
rs9398196
rs1985369
rs11584821
rs1907702
rs28363070
rs11412296
rs117034449
rs116863223
rs10905255
rs6054257
rs9600019
rs2230590
rs34550383
rs4731541
rs7099229
rs141214782
rs3093888
rs76666449
rs450962
rs12369950
All − MR Egger
All − Inverse variance weighted
−2
−1
0
1
2
MR effect size for
'telomere length || id:ieu−b−4879' on 'IDP_dMRI_TBSS_MD_Posterior_corona_radiata_
